# Biosensor Technology Reveals the Disruption of the Endothelial Barrier Function and the Subsequent Death of Blood Brain Barrier Endothelial Cells to Sodium Azide and Its Gaseous Products

**DOI:** 10.3390/bios7040041

**Published:** 2017-09-21

**Authors:** Dan T. Kho, Rebecca H. Johnson, Simon J. O’Carroll, Catherine E. Angel, E. Scott Graham

**Affiliations:** 1 Department of Pharmacology and Clinical Pharmacology, School of Medical Sciences, Faculty of Medical and Health Sciences, University of Auckland, Auckland 1010, New Zealand; d.kho@auckland.ac.nz (D.T.K.); rebecca.johnson@auckland.ac.nz (R.J.); 2 Centre for Brain Research, University of Auckland, Auckland 1010, New Zealand; s.ocarroll@auckland.ac.nz; 3 Department of Anatomy and Medical Imaging, School of Medical Sciences, Faculty of Medical and Health Sciences, University of Auckland, Auckland 1010, New Zealand; 4 School of Biological Sciences, Faculty of Science, University of Auckland, Auckland 1010, New Zealand; c.angel@auckland.ac.nz

**Keywords:** sodium azide, ECIS, brain endothelium

## Abstract

Herein we demonstrate the sensitive nature of human blood-brain barrier (BBB) endothelial cells to sodium azide and its gaseous product. Sodium azide is known to be acutely cytotoxic at low millimolar concentrations, hence its use as a biological preservative (e.g., in antibodies). Loss of barrier integrity was noticed in experiments using Electric Cell-substrate Impedance Sensing (ECIS) biosensor technology, to measure endothelial barrier integrity continuously in real-time. Initially the effect of sodium azide was observed as an artefact where it was present in antibodies being employed in neutralisation experiments. This was confirmed where antibody clones that were azide-free did not mediate loss of barrier function. A delayed loss of barrier function in neighbouring wells implied the influence of a liberated gaseous product. ECIS technology demonstrated that the BBB endothelial cells had a lower level of direct sensitivity to sodium azide of ~3 µM. Evidence of gaseous toxicity was consistently observed at 30 µM and above, with disrupted barrier function and cell death in neighbouring wells. We highlight the ability of this cellular biosensor technology to reveal both the direct and gaseous toxicity mediated by sodium azide. The sensitivity and temporal dimension of ECIS technology was instrumental in these observations. These findings have substantial implications for the wide use of sodium azide in biological reagents, raising issues of their application in live-cell assays and with regard to the protection of the user. This research also has wider relevance highlighting the sensitivity of brain endothelial cells to a known mitochondrial disruptor. It is logical to hypothesise that BBB endothelial dysfunction due to mitochondrial dys-regulation could have an important but underappreciated role in a range of neurological diseases.

## 1. Introduction

The brain is a highly vascularised organ where the brain microvascular endothelial cells sub-serve an incredibly important function maintaining normal brain homeostasis, including the brain’s nutrient supply. Additionally, the brain endothelial cells maintain a selective barrier to peripheral immune cells, whereby the vast majority of peripheral leukocytes are actively excluded from the central nervous system, except during states of neuroinflammation. Brain endothelial cells are polarised with their apical aspect in contact with the blood and their baso-lateral membrane juxtaposed to pericytes and astrocytic end-feet; this unit is collectively referred to as the neurovascular unit [1,2,3]. Importantly, with regard to the brain endothelium, they express high levels of tight junction proteins and adherens junction molecules, which collectively confer a higher level of barrier tightness in comparison to other vascular beds throughout the body.

The most advanced toolbox for measuring the real-time barrier integrity of endothelial cells in vitro is the Electric Cell-substrate Impedance Sensing (ECIS) cellular biosensor [4,5,6,7]. ECIS measures the flow of current between opposing electrodes imbedded in custom ECIS plates, where a strong endothelial barrier will confer a greater resistance to the flow of the electrons in comparison to a weak barrier [4,8,9]. ECIS measures barrier function in real-time and in a label free manner, and allows for both acute and longer term assessment of a variety of treatment paradigms (including inflammatory activation, the addition of leukocytes, drug treatments, and many more). In this regard, we have been using ECIS technology extensively to assess the temporal effects of various drugs [7], immune cells [10], and other inflammatory mediators on brain endothelial barrier function [11].

During a study investigating the involvement of specific cell-surface leukocyte adhesion molecules and their temporal role in the engagement of activated leukocytes (e.g., diapedesis), we inadvertently observed the toxic effects of sodium azide where it was present as a preservative in a range of antibodies. Sodium azide has been found to induce cytotoxicity in neuroblastoma in the high µM to mM range, potentially through disrupting mitochondrial complex IV activity and mitochondrial membrane potential [12]. Furthermore, the blood-brain barrier endothelial cells are also highly susceptible to toxic substances such as cyclodextrins [13], malathion [14] and aflatoxins [15]. Here, we have made the discovery that human brain endothelial cells [7,11] are sensitive to sodium azide when barrier compromise occurs in the low µM range, which is more than 100 fold lower than we would have expected. In addition, we reveal the unexpected toxicity of a gaseous product, which had a detrimental effect on the viability of cells in neighbouring untreated wells.

In this paper, we highlight the incredible power of ECIS biosensor technology to reveal the temporal effects of sodium azide and the inadvertent observation that sodium azide liberated a gaseous product that was highly toxic to neighbouring untreated cells. We suspect that brain endothelial cells are particularly sensitive to sodium azide because of the innate high-energy demand required to maintain their molecular gradients and barriers. Our observations have important implications for the field of neuroscience, clinical neurology, and the azide’s broad spectrum application as a preservative in many antibody-based products.

## 2. Materials and Methods

Standard cell culture reagents were purchased from Invitrogen and Gibco unless otherwise specified. Sodium azide was purchased from Sigma (Cat# S2002). The antibodies used in the ECIS experiments were purchased from Biolegend (San Diego, CA, USA). Where available, LEAF clones (low endotoxin azide-free) were also purchased from Biolegend. ECIS plates were purchased from Applied Biophysics (Troy, NY, USA).

### 2.1. Cell Culture of hCMVECs

The human cerebral microvascular endothelial cell (hCMVEC) was purchased from ABMGood (Cat# T0259). Cells were maintained in M199 media (Cat# 11150067, Gibco, Waltham, MA, USA), supplemented with 10% FBS (Moregate Biotech, Bulimba, QLD, Australia), 1 µg/mL hydrocortisone (Cat# H0888, Sigma, St. Louis, MO, USA), 3 ng/mL human FGF (Cat# PTAF10018B50, PeproTech, Rocky Hill, CT, USA), 1 ng/mL human EGF (Cat# PTAF10015100, PeproTech), 10 µg/mL heparin (Cat# H3393, Sigma), 2 mM Glutamax (Cat# 35050-061, Gibco), and 80 µM di-butyryl cAMP (Cat# D0627, Sigma); referred to as M199 10% growth media. Cells were typically used under passage 20 with media being replenished every 2–3 days as required.

### 2.2. Barrier Integrity Measurements Using ECIS ZΘ Technology

ECIS experiments were conducted using 96W20idf ECIS arrays in conjunction with the ECIS ZΘ station, as described previously [7,11]. In brief, wells were prepared by coating with 10 mM cysteine (Cat# C7352, Sigma) followed by coating with 1 µg/mL collagen I (Cat# A1048301, Gibco) giving 1 µg/cm² (as per manufacturer’s recommendation). After collagen coating, the arrays were placed in the ECIS array station to ensure the electrodes were properly connected and had not been damaged during the coating procedure. Following array preparation and electrode assessment, the hCMVECs were harvested from a confluent T75 flask using 0.05% trypsin-EDTA (Cat# 25300120; Gibco). Cells were used between P7 and P19 and seeded at 54,000 cells in 100 µL of M199 10% growth media.

ECIS was conducted using the single frequency option at 4000 Hz, which is the recommendation for measuring barrier resistance. Cells were typically treated at 24 or 48 h after seeding when a strong barrier had formed. This was determined previously as the period where the barrier strength level had stabilised (700–900 Ω) [7,11]. Drugs were added to respective wells as 2× concentrates from donor 96 well plates. Treatments were conducted in at least triplicate. Following drug addition, the ECIS experiments were continuously monitored for 4–5 days to capture both acute and longer term changes in barrier resistance.

### 2.3. ATP Measurement for Assessment of Cell Viability

The hCMVECs were harvested from a confluent T75 flask using 0.05% trypsin-EDTA. Cells were seeded into collagen-coated 96 well plates at 54,000 cells in 100 µL of M199 10% growth media. Cells were then treated approximately 48 h after seeding for the designated period, as indicated in the figure legend. In these assays, cellular content of ATP was measured, therefore culture media (which may contain released or liberated ATP) was removed prior to lysis of the cells. ATP levels were measured using the ATPlite Luminescence Assay System (Cat# 6016943, Perkin Elmer, Waltham, MA, USA) following the manufacturers protocol. The resultant luminescence was measured using a VictorX light 2030 Luminescence reader (Perkin Elmer), and data were graphed using Prism version 7.

### 2.4. Cell Imaging

For imaging, the cells were seeded at 54,000 cells in 100 µL of M199 10% growth media in 96-well plates and were grown for 48 h. Sodium azide was added as 2× concentration to achieve a final concentration of 0.0002%. Control wells received media only. Bright field images of live cells were acquired using an EVOS FL Auto Cell Imaging System (Thermofisher, Waltham, MA, USA) at 72 h and 96 h post treatment.

### 2.5. Statistics

Data are presented graphically as mean ± SEM for data from one representative experiment. Experiments were repeated at least 3 times. Statistical analyses were performed using GraphPad Prism^®^ 6.07 (GraphPad Software Inc., LaJolla, CA, USA). One-sample T tests were performed to determine the statistical differences between the cell viability of treated cells and control cells.

## 3. Results

### 3.1. Antibody Preparations Containing Sodium Azide Mediate Delayed Loss of Endothelial Barrier Resistance, as Measured Using ECIS Technology

**Figure 1:**
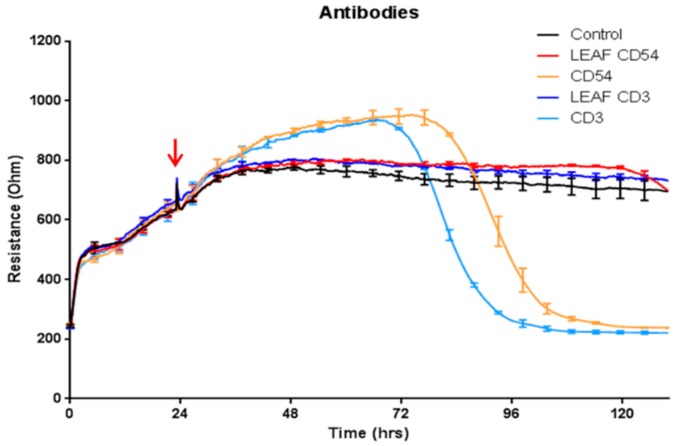
Commercial antibodies containing sodium azide induce loss of brain endothelial cell (BEC) barrier integrity. Antibodies direct to CD54 and CD3 were added to the BECs at the time point demarcated by the red arrow. The CD54 and CD3 antibodies that contained azide (stock 0.09%) are shown by the light blue and orange curves. Final sodium azide concentration (*v*/*v* %) in the treatment was 0.0009%. The respective LEAF (low endotoxin azide free) versions for CD54 and CD3 antibodies are dark blue and red, respectively. The control media treated BECs are shown by the black curve. These data have been repeated at least five times and are representative of at least 15 different antibody preparations that contain sodium azide. Curves show the mean ± SEM (*n* = 4 Electric Cell-substrate Impedance Sensing (ECIS) wells).

The observation that brain endothelial cells (BECs) are highly sensitive to sodium azide was made whilst conducting leukocyte trafficking experiments. Initial experiments saw pronounced ‘antibody’ mediated loss of barrier integrity 2–3 days after initial addition of the diluted antibodies (diluted ~1:100). Figure 1 summarises these experiments, comparing antibody clones for both the LEAF (low endotoxin azide free) versions of the antibody to the azide containing equivalent clone. It is clear from these data that the pronounced loss in barrier integrity was not evident with the azide free antibodies. An additional range of sodium azide containing (0.05% to 0.08%) antibodies were diluted in the ECIS experiments where the sodium azide concentration was approximately 0.0002–0.00005% (see Table 1 for the molarity conversion). Every antibody containing azide affected the barrier integrity in a similar manner to that shown in Figure 1, irrespective of antibody preparation and target.

### 3.2. BBB Endothelial Cells are Highly Sensitive to Sodium Azide

**Figure 2:**
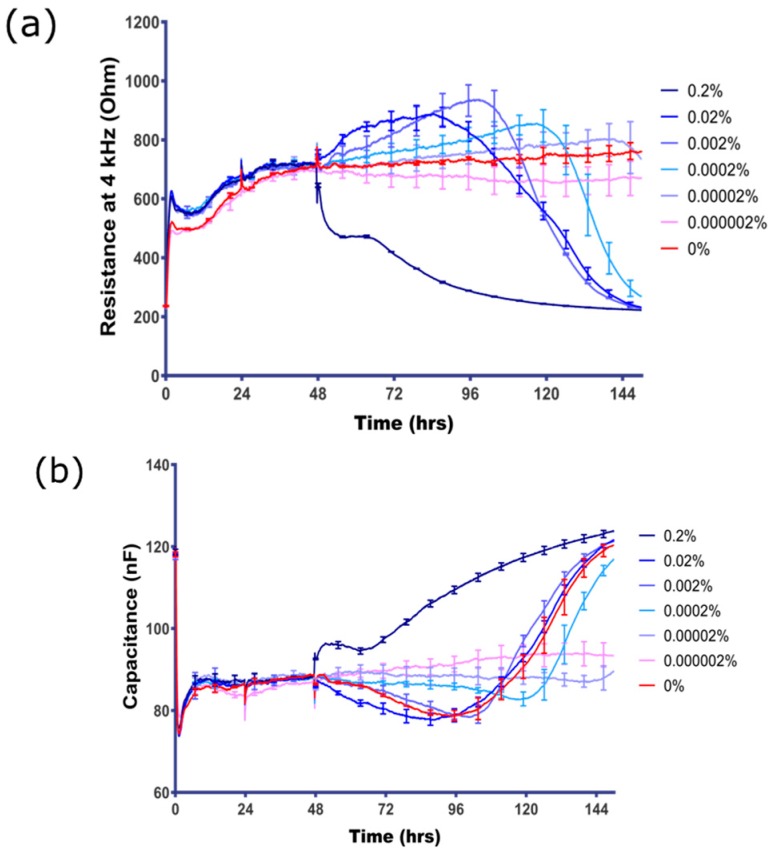
Direct assessment of sodium azide concentration response on BEC. (**a**) The resistance of BEC measured at 4 kHz represents the barrier integrity; (**b**) the electrode capacitance of BEC is inversely related to the surface coverage of the electrode by the endothelial cells. Final concentration of sodium azide (range 0–0.000002%) is shown in the colour-coded figure key. Note that the control media-only treatment is the red curve, which shows a very stable barrier for the duration of the time course. These data show mean ± SEM (*n* = 4 wells) and are representative of at least five independent observations.

The literature demonstrates that sodium azide is acutely cytotoxic at concentrations in the high micromolar (µM) to millimolar (mM) range [12,16,17], hence its common use as a preservative agent in most antibody products and some other laboratory products (see Table 2). Whilst acute toxicity in this range was expected, Figure 2 demonstrates that the hCMVECs are extremely sensitive to sodium azide and reveals an intriguing temporal profile of sensitivity, with toxicity noted as low as 2 × 10^−6^% sodium azide (Figure 2a,b). Interestingly, acute or immediate loss of barrier integrity was only observed at the highest concentrations of azide (>0.02%); whereas concentrations below 0.02% mediated loss of barrier integrity but after a delayed period (>24 h). The loss of integrity was observed with cells treated with sodium azide concentrations as low as 2 × 10^−7^% (30 nM) (Figure 2a,b). It is worth noting that ECIS technology reveals a pronounced increase in barrier strength prior to the rapid barrier failure. This increase can be observed prominently for sodium azide at 0.02% and 0.002% (3 mM and 300 µM) when there is an increase in barrier strength for 40–50 h after azide addition. However, after this increase, the barrier integrity is lost rapidly and resistance reduces to 200 Ω, which is the resistance in the absence of cells. Along with the increase in electrode capacitance (Figure 2b), this indicates that the endothelial barrier integrity is lost completely and the cells no longer cover the electrode. The latter is indicative of cell-death.

### 3.3. Sodium Azide Liberates a Gaseous Product that Affects the Endothelial Barrier Integrity

**Figure 3:**
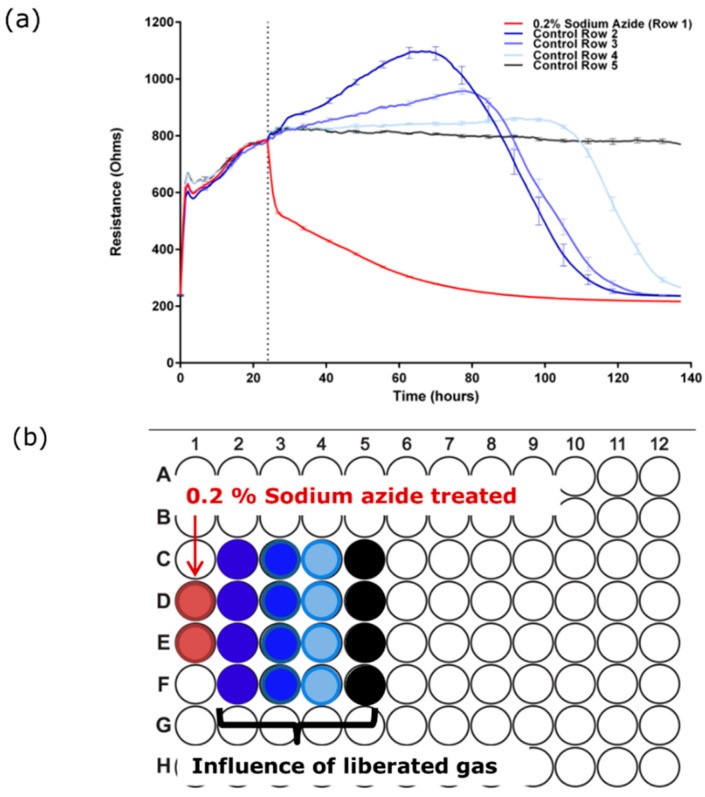
Sodium azide forms a gaseous product which also destroys the barrier integrity of the BECs. (**a**) Sodium azide (0.2%) was added to the far left wells of an ECIS 96 well plate and control media was added to the rest of the plate. Gaseous liberation affects cells 3 wells distal to the site of addition; (**b**) plate map shows location of treated wells relative to data in (**a**). These data have been replicated independently at least six times.

Whilst conducting the pharmacological concentration response experiments (Figure 2), we noticed a pronounced loss of the barrier integrity of untreated cells in the wells neighbouring the 0.2% sodium azide treatment. This observation implied liberation of a gaseous product from the azide-treated well. In a subsequent experiment wells in row 1 of an ECIS plate were treated with culture media containing 0.2% sodium azide and the remainder of the wells received media only. Figure 3 reveals that the gaseous product liberated from the 0.2% sodium azide treated-well affected the barrier strength in cells up to 3 wells distal to the treated wells. This confirmed that there was both a direct effect of the sodium azide and a potential influence of a gaseous product. However, the discovery of the gaseous product could potentially complicate the interpretation of the data presented in Figure 2.

### 3.4. Determining the Barrier Integrity Loss Due to Direct Effect of the Sodium Azide vs the Gaseous Product

**Figure 4:**
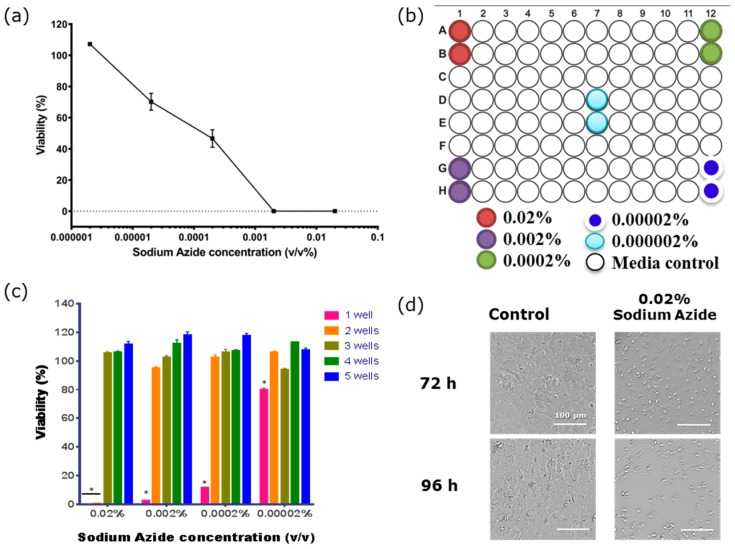
The viability of BECs is affected by both sodium azide and its gaseous product. (**a**) Measurement of sodium azide toxicity measured using cellular adenosine triphosphate (ATP) levels to measure cellular viability. Vehicle-treated cells represent 100%. Viability was measured 96 h after exposure to the sodium azide. (**b**) Plate map shows the position of the sodium azide-treated wells. (**c**) ATPlite assay reveals the toxic effect of the gaseous product produced from the sodium azide on the cells in the wells neighbouring the directly treated wells at 120 h. One Sample T tests show that cells 1 well away are significantly affected by the gaseous toxicity (* *p* < 0.0001). (**d**) Bright field images of hCMVECs 72 h (top panels) and 96 h (bottom panels) post-treatment with 0.02% sodium azide (right panels) or media only (left panels). Data shows mean ± SEM of two wells from of a single experiment, which is representative of three independent observations.

As the distal effect of the azide gas seemed to be limited to 3 wells or less, the plate map in Figure 4b was used to determine: (i) the lowest concentration of azide that had a direct effect on endothelial barrier function; and (ii) which conditions produced the toxic gaseous product (see summary data in Table 3). The data shown in Figure 4a reveals that the direct effect of sodium azide on the barrier compromise was observed at concentrations as low as 2 × 10^−5^%. The azide gas was also produced from the wells treated with sodium azide at 2 × 10^−4^% and above (Figure 4c). In addition to measuring endothelial resistance using ECIS technology we also directly measured cellular ATP levels at 120 h post-treatment as a function of cell health and viability (Figure 4). These results confirmed the ECIS data presented above and demonstrated that the loss in resistance was associated with the compromise and loss of endothelial cells. Bright field imaging confirmed that the majority of the azide-treated cells were lost 72 h post treatment (Figure 4d).

## 4. Discussion

Herein we demonstrate the sensitive nature of human brain endothelial cells to sodium azide, whereby irreversible loss of endothelial barrier integrity was used as the measure of azide toxicity. Brain endothelial cells maintain cerebral homeostasis due to high levels of tight junction complexes [18,19] and a range of ATP-driven transporters [20,21,22]. These mechanisms place a high energy demand on the endothelial cells, perhaps explaining the azide sensitivity. However, the level of sensitivity and the production of a gaseous toxicant were unexpected. The subsequent barrier disruption in neighbouring wells was followed by cell death, indicating sodium azide has toxicity in the low micromolar range, ~100 fold lower than the cytotoxic effects observed by others [12,16,17].

The concentration of sodium azide used as a preservative in antibodies and other reagents (~0.05 to 0.1%) had an immediate effect on barrier function and viability. However, at lower azide concentrations, the effect on barrier integrity required several days to manifest. This response is interpreted as a delayed cytotoxic effect, in which the endothelial cells initially strengthened their barrier. This is a particularly interesting observation, which suggests that the endothelial cells detected the azide as a danger signal and responded accordingly by innately strengthening the barrier. However, after this period, the loss of barrier integrity was rapid and permanent. At the lowest concentrations of azide, this did not occur until at least 90–100 h after addition to the cells. Part of the cytotoxic mechanism of action was the liberation of a gaseous product, which influenced the barrier integrity of the endothelial cells in wells immediately adjacent to the directly treated wells in the ECIS plate. These adjacent wells had only been treated with control media and had not been exposed directly to sodium azide. This may implicate hydrazoic acid (see review [23]), which is highly volatile and produces a gas at 37 °C.

Sensitivity of ECIS to screen for BBB disrupting factors—These observations exemplify the power of ECIS technology to measure endothelial barrier function, in this context barrier integrity. Thus, the temporal nature of the ECIS measurements, specifically the ability to measure integrity continuously for days, has aided our observations of the sensitivity of these cells to sodium azide. Not only were we able to monitor the temporal response over ~5 days, but ECIS is sufficiently sensitive to detect small changes in barrier strength (e.g., the acute increase in barrier strength prior to the permanent loss). This may not have been observed had we simply assessed viability after several hours using a classical viability stain (e.g., 7AAD used during flow cytometry) following isolation or treatment of the cells. It is very possible that some cell types will not be sensitive to the sodium azide at the lower concentrations used here and that this sensitivity maybe a function related to the energy demand of brain endothelial cells or other facets relating to the biology of brain endothelial cells.

Biomedical application of products containing ‘low levels of azide’—It is common knowledge amongst researchers that many laboratory products (see Table 2) contain sodium azide, typically at 0.05–0.08%, as a bactericidal and fungicidal agent. This includes primary antibodies, secondary antibodies, and, perhaps more importantly, various cell isolation kits from a range of manufacturers. For the latter, the sodium azide is present in the antibody cocktails and/or buffers, whereby the living cells come into contact directly with these components during the labelling, washing steps, and isolation steps, meaning they could be exposed to the azide for several hours depending on the protocol. The primary concern we wish to highlight is the underappreciated effect the sodium azide would have on the living cells, especially cells used for long term culture, differentiation, or where patient cells are prepared for clinical applications (e.g., transplant). This would occur, for example, with isolation kits or following FACS of cells, where the cells could be maintained in culture for weeks or longer. The solution here is relatively simple for the direct use of antibodies, where a LEAF variant is available. Alternatively, antibodies can be purified using molecular weight filtration columns (e.g., Microcon 10–30 kDa filters), which are widely available. The solution is more complicated when using proprietary kits where the components of the antibody cocktails or buffers may not be known. This represents a challenge in terms of removing the azide, as there will also be inherent loss of antibodies during the filtration steps.

An important clinical and biomedical consideration is for the preparation of a patient’s cells for applications, including: (i) homologous stem cell transplantation; (ii) immunotherapy applications, where a patient’s immune cells may be manipulated to enhance immune responses (e.g., antigen presentation or T cell responses); and for (iii) tissue engineering (e.g., generating replacement skin for burns victims). Regardless of the clinical avenue, serious consideration should be given to the reagents used to isolate and prepare these cells as ultimately they will be delivered back into the patient. For immunological reasons the use of ‘low-endotoxin’ reagents has been recommended for years; now, awareness needs to extend to the sodium azide too.

## 5. Conclusions

Other sources of azide—We were well aware of the azide present in laboratory products, but are less aware of the other uses of sodium azide (see Table 2). For instance, we were extremely surprised and now more than a little concerned that vehicular airbags contain kilogram quantities of sodium azide, which is used to inflate the bag. These are obviously designed to deploy during a car crash and under normal conditions result in the formation of nitrogen gas. However, concern is being voiced about the growing environmental risk of airbags present in wreckage yards, land fill sites, and the potential risk of soil and ground water contamination. Further detailed information on this can be sourced from the CDC (see http://www.bt.cdc.gov/agent/sodiumazide/basics/facts.asp). This also has clinical relevance where a person in a car accident or car fire may be exposed to toxic levels of sodium azide or hydrazoic gas, and this may go un-considered in the emergency department, especially where the focus may be on a resultant traumatic brain injury or spinal injury.

Clinical implications of energy disruption in endothelial vessels in the brain and association with neurological diseases—The human brain is highly vascularised, and marked vascular disease is noted in a range of neurological conditions [24,25,26,27,28,29,30,31]. The data presented here raises the consideration that processes (degenerative, disease, infection, or cytotoxic) that affect the mitochondrial function or the energy homeostasis of cells such as the vascular endothelial cells in the neurovascular unit of the brain would have serious implications for long term brain health and may lead to blood-brain-barrier compromise or loss of blood vessels in the brain.

## Figures and Tables

**Table 1:** Sodium azide percentage to molarity conversion.

Sodium Azide (%)	Sodium Azide Molarity
**0.2%**	30 mM
**0.02%**	3 mM
**0.002%**	300 µM
**0.0002%**	30 µM
**0.00002%**	3 µM
**0.000002%**	300 nM
**0.0000002%**	30 nM

**Table 2:** List of laboratory products containing sodium azide.

Product	Azide Present	Comments
Primary antibodies	0.05 to 0.1% 7.5 mM to 15 mM	Majority of primary antibodies where storage is at 4 °C will contain azide
Secondary antibodies	0.05 to 0.1%	Majority of antibodies where storage is at 4 °C will contain azide
Flow cytometry antibodies	0.05 to 0.1%	Some LEAF versions are now available from certain vendors
Cell isolation kits (e.g., Miltenyi)	0.05 to 0.08%	Antibody cocktails and some buffers contain azide
ELISA kits	0.05–0.1%	Storage buffers containing azide; Disposal issues
Other assays kits involving buffers and antibody storage	0.05–0.1%	Storage buffers containing azide; Disposal issues
Vehicle airbags	kg quantities	Issues with exposure of the azide resulting in hydrazoic gas formation.Long term environmental effects not known
Aircraft escape shuts	>100 kg quantities	

**Table 3:** Summary of the direct and gaseous toxicity of sodium azide on brain endothelial cells.

Sodium Azide Concentration	Direct Toxicity	Gaseous Toxicity
0.02%	Yes	Yes
0.002%	Yes	Yes
0.0002%	Yes	Yes
0.00002%	Yes	Minimal
0.000002%	No	No
0.0000002%	No	No
